# Career choice, training satisfaction, and retention intentions among healthcare apprentices: a cross-sectional study in rural Germany

**DOI:** 10.1186/s12913-026-15022-y

**Published:** 2026-07-07

**Authors:** Andree Ehlert, Dirk Oberschachtsiek

**Affiliations:** Harz University of Applied Sciences, Friedrichstr. 57 – 59, 38855 Wernigerode, Germany

**Keywords:** Nursing education, Internships, Career motivation, Personnel retention, Rural healthcare, Demographic change

## Abstract

**Background:**

Workforce shortages pose a significant challenge to healthcare provision across Western countries, making the motivations and retention of future professionals a priority for research and policy.

**Methods:**

This study investigates key factors influencing career decisions and training experiences among apprentices in generalist nursing and special needs care, based on 242 standardized surveys conducted in nine classes at a health and social care training center in rural Germany. The study specifically investigates what motivates apprentices’ career choices, the expectations they hold upon entering training, and how early experiences in vocational school and in-firm training influence their intention to remain in the profession.

**Results:**

Our findings indicate that traditional media exposure has minimal influence on career choice, whereas proximity and geographic accessibility emerge as key criteria. Importantly, negative experiences in vocational school curricula and inadequacies in firm-based training are significantly associated with reduced commitment to stay in the profession.

**Conclusions:**

Targeted interventions addressing curricular content, pedagogical support, and coordination between school and field training are crucial to reducing workforce attrition in regional healthcare.

**Supplementary Information:**

The online version contains supplementary material available at 10.1186/s12913-026-15022-y.

## Introduction

Health systems face mounting workforce pressures as populations age and care needs rise. In Germany, approximately six million individuals — around 13% of the total workforce — are employed in healthcare. Despite a 20% increase in healthcare employment over the past decade, projections indicate a substantial shortfall in meeting future demand. According to the Pflege-Report 2023, an additional 500,000 healthcare professionals will be required by 2030, partly due to an 80% increase in care-dependent individuals — from 2.3 million in 2012 to over 4.1 million in 2021 [42]. Long-term care is particularly affected by this demographic shift, as the number of people aged 80 and above continues to grow. The 2024 Advisory Council Report further forecasts a gap of 280,000 to 690,000 nursing professionals by 2049, underscoring the critical urgency of attracting and retaining young talent within healthcare professions [39].

Workforce shortages are not spatially neutral. Rural and peripheral regions combine smaller and more dispersed training markets, longer travel times to vocational schools and firms, and fewer nearby clinical placements. These structural features can amplify the frictions that apprentices encounter at the school–work interface and may shape both entry and early retention decisions. Saxony-Anhalt, the setting of this study, exemplifies such dynamics: it is predominantly rural, with a population density of only 107 inhabitants per square kilometer (as of 2022), ranking it as the federal state with the third-lowest density, less than half the German national average. Furthermore, population ageing is comparatively advanced, intensifying the demand for care services (Statistisches Landesamt Sachsen-Anhalt, [46]).

Despite the urgency of workforce challenges — particularly in rural contexts — there is limited empirical evidence on what drives apprentices’ career decisions and retention intentions. Most quantitative research focuses either on fully qualified professionals or on urban training markets and aggregated staffing trends [4, 31, 37], thus overlooking the specific dynamics and spatially conditioned frictions inherent in rural training ecosystems. Public discourse often assumes widespread dissatisfaction among trainees, yet systematic statistical validation of these claims in rural regions remains scarce. Our study addresses this gap by providing a rigorous quantitative analysis of motivational, experiential, structural, and organizational determinants affecting apprentices in nursing and special needs care within a rural German setting.

The overarching research question of this study is: *What drives career choice*,* training satisfaction*,* and retention intentions among healthcare apprentices in rural Germany?* This main question is operationalised through four sub-questions that structure the empirical analyses in Sections “Motivational influences on career choice” to Determinants of future retention intentions: (**RQ1**) Which motivational factors are most important for the choice to enter healthcare training? (**RQ2**) How do prior internship experience and the geographic accessibility of the training institution shape training decisions? (**RQ3**) How do apprentices evaluate the school-based and company-based components of their training, and do these evaluations relate to career intentions? (**RQ4**) What determines the intention to stay in the specific occupation and in the broader healthcare sector? These four research questions are grounded in established career-development theory. Our empirical strategy draws together three complementary lenses: Self-Determination Theory [9, 38], which frames the analysis of career-choice motivation; Social Cognitive Career Theory [29, 30], which structures the treatment of contextual barriers and of training-stage learning experiences; and the push-pull framework widely used in health-workforce research [12, 28], which informs the analysis of the sector-versus-occupation retention differential. Section “Theoretical Grounding” develops this theoretical grounding and maps the constructs onto the specific analyses that follow.

To address these research questions, we apply a combination of descriptive and inferential statistical techniques, including factor and cluster analyses, non-parametric group comparisons, correlation analysis, and ordinal regression models. Data collection took place in February and March 2025 across nine classes at a health and social care training center in Saxony-Anhalt. The survey was administered in-class via QR code and survey link during scheduled lessons, yielding 242 valid questionnaires. While the sample is not representative of the entire German healthcare training sector, it deliberately focuses on a rural region particularly affected by demographic change and population decline, where the challenges outlined above are especially pressing. Similar structural conditions exist in many parts of Germany [13], making the findings of broader relevance.

The remainder of this article is structured as follows: Section “Conceptual framework” situates our study within the relevant literature and theoretical frameworks. Section “Data and methods” describes the data collection, questionnaire design, and empirical strategy. Section “Results” presents the results, organised along RQ1 to RQ4. Section “Discussion” discusses their implications for policy and practice. Section “Conclusion” concludes.

## Conceptual framework

### Theoretical grounding

The empirical determinants reviewed in Section “Determinants of career retention and career decisions in nursing and healthcare apprenticeships” below are embedded in a theoretical framework that combines three complementary lenses, each with a distinct analytical role. Taken together, they allow the empirical findings that follow to contribute to an established theoretical conversation rather than remaining purely descriptive patterns.

Self-Determination Theory (SDT; [9, 38]) distinguishes autonomous motivation — grounded in intrinsic interest, identified regulation, and integrated regulation — from controlled motivation, which is sustained by external contingencies or introjected pressures. Applied to vocational and work settings, autonomous motivation is systematically associated with higher persistence, deeper learning, and psychological well-being, whereas controlled motivation can produce short-term behavioural compliance but not sustained engagement [16]. In the present study, SDT provides the primary lens for the seven career-choice motivational items analysed in Sections “Motivational influences on career choice”: personal interest, prior internship experience, and proximity to home represent paradigmatic autonomous drivers; media, career counselling, and parental advice paradigmatic controlled drivers; income prospects occupies an intermediate, instrumental position between the two poles.

Social Cognitive Career Theory (SCCT; [29, 30]) models career choice and persistence as a joint function of self-efficacy beliefs, outcome expectations, and the contextual supports and barriers that surround them. SCCT is especially useful in this context because its contextual branch formalises the role of proximal environmental factors — commuting burden, family-care obligations, geographic accessibility — that our transport and accessibility findings (Section “Internship experience and geographic accessibility”) foreground. Equally, its learning-experience pathway frames the school- and company-based training evaluations (Section “Practical experiences and orientation”) as the proximal inputs through which vocational training builds, or erodes, the self-efficacy beliefs and outcome expectations that ultimately feed persistence in training and beyond (Sections “Determinants of future retention intentions”).

Push-pull frameworks of health-workforce attraction and retention [12, 28, 53] complement these individual-level theories with an explicit vocabulary for structural factors that destabilise attachment to a specific occupation (push) and for those that attract or retain personnel within a given labour market segment (pull). This lens is especially productive for the sector-versus-occupation retention differential that our two-item retention design (Sections “Determinants of future retention intentions”) makes visible: apprentices may experience occupation-specific push while remaining anchored to the wider healthcare sector by lateral pull toward adjacent roles. In rural contexts, push factors (longer commutes, thinner labour markets, fewer specialised placements) tend to concentrate while within-region pull factors are scarcer, rendering rural retention structurally harder than urban retention. Although this evidence base stems largely from middle- and low-income countries, the underlying mechanisms of distance, labour-market thinness, and scarce placements apply equally to peripheral regions of high-income countries [28].

Holland’s RIASEC typology of vocational fit [20] is acknowledged for completeness but not adopted as an analytical axis here: our sample is drawn from apprentices who have already self-selected into a narrow slice of the Social interest code, and the generalist-nursing-special-needs-care distinction yields insufficient typological variance to generate incremental insight beyond what SDT and SCCT capture. The operational mapping of theoretical constructs onto specific survey items is developed in Section “Questionnaire development”.

### Determinants of career retention and career decisions in nursing and healthcare apprenticeships

Recent literature identifies a broad spectrum of factors influencing the retention of nursing and healthcare professionals, conventionally grouped into personal, organisational, and contextual determinants. Within this dimensional organisation, four substantive themes are particularly relevant to our research questions: motivational influences and organisational support (RQ1); practical experiences at the school-work interface (RQ2, RQ3); individual heterogeneity and evolving career perceptions (RQ3); and structural and organisational conditions in rural training ecosystems (RQ2, RQ4).

Motivational influences and organisational support. Schmedding et al. [40], Sommer et al. [44], and Pressley and Garside [35] consistently link career entry and long-term professional stability to personal competencies, task integration, social appreciation, and organisational support, including coping resources for occupational stress.

Practical experiences and the school-work interface. The transition from vocational schooling to professional practice is a critical phase of occupational socialisation, shaped in Germany by the distinctive structure of vocational nursing education, which in broad terms shares features with dual training systems in Austria and parts of Switzerland while remaining specific in the division of responsibilities between vocational schools and care facilities. Challenges at this interface typically arise from divergent learning objectives, inconsistent competency recognition, and varying expectations between learning environments [10, 17, 33, 50]. Effective onboarding [10] and practitioners’ ability to accommodate heterogeneity in apprentices’ prior knowledge [17, 33] are key determinants of retention at this transition.

Individual heterogeneity and evolving career perceptions. Apprentices differ markedly in how training shapes their career outlook. Garcia-González and Peters, [17] identify male and younger apprentices as groups at elevated risk of training discontinuation, while De Vries et al. [10] report that the influence of gender on retention is inconsistent across studies. Review evidence further indicates that younger, early-career nurses are more transient and less attached to their organisations than older colleagues [35]. Koob and Tomic [26] further show that the perceived personal significance of healthcare work — their “ikigai” measure — correlates positively with work engagement during training, a finding coherent with the SDT prediction that autonomously regulated work yields stronger persistence than externally regulated engagement [16].

Structural and organisational conditions in rural training ecosystems. Locality and accessibility shape retention especially in rural areas. King et al. [25] and Drange and Ingelsrud [11] identify local labour-market thinness and perceived post-training career options as decisive drivers of professional retention; Sommer et al. [44] emphasise predictable schedules and sufficient direct patient contact. Read through SCCT’s contextual branch and the push-pull lens, rural training ecosystems concentrate push factors (longer commutes, thinner labour markets, fewer specialised placements) while offering fewer within-region pull factors, rendering rural retention structurally harder than its urban counterpart [28, 29].

The present study addresses these four themes in the rural German context along the research questions introduced in Section “Introduction”; the construct-to-item mapping that operationalises them is set out in Section “Questionnaire development”.

## Data and methods

### Research design and sample

This study employed a cross-sectional survey design, with a particular focus on rural settings in the Nordostharz region of Germany. The target population comprised all apprentices currently enrolled in generalist nursing and special needs care (“Generalistische Pflegeausbildung” and “Heilerziehungspflege”) at a health and social care training center, which serves the catchment area of Wernigerode, Halberstadt, Neinstedt, and Elbingerode.

Participants were recruited via school-based communication channels in close collaboration with teaching staff. Both paper-based questionnaires and online survey links were distributed, maximizing accessibility and inclusivity within the school environment. Of the 242 valid responses, 100 were submitted online and 142 on paper. Data collection was conducted during scheduled lessons at the training center between early February and March 2025. All classes pertaining to the two target vocational tracks were invited to participate, reflecting an inclusive approach with minimal exclusion criteria beyond enrolment status. All apprentices present in the invited classes on the survey days returned valid questionnaires, corresponding to a unit-level response rate of 100% among those present; absentees on a given day (e.g., due to illness or scheduled internships) are accordingly not represented in the dataset. Item-level missingness varies across analyses; complete-case sample sizes are reported alongside each statistical test in Sections “Motivational influences on career choice” to “Determinants of future retention intentions”.

Ethical approval was obtained in advance, and participants provided explicit information and self-declaration concerning data privacy in accordance with current legal and institutional requirements, and their right to withdraw at any time. The survey setting and timing, embedded within regular class activities at the training center, offered a familiar environment while ensuring that responses reflected current educational experiences. The item “prior internship” refers to any pre-apprenticeship internship undertaken as part of career orientation, regardless of sector; this operationalization is retained throughout the analyses.

Table 1 reveals a predominantly young and educationally homogeneous cohort: almost 50% of apprentices are 20 years or younger (38% aged 18–20; 11% under 18), whereas only 10% are over 30. The gender split is essentially balanced (49% male, 47% female), and the great majority (69%) hold an intermediate secondary certificate, with almost half (47%) reporting prior work experience. Daily round-trip commuting averages roughly 35 min to both workplace and vocational school.

**Table 1:** Sample characteristics

		*n*	%	mean	sd
Age	< 18	27	11.2		
	18-20	92	38.0		
	21-25	64	26.5		
	26-30	18	7.4		
	> 30	23	9.5		
	NA	18	7.4		
Gender	male	119	49.2		
	female	114	47.1		
	non-binary	0	0.0		
	NA	9	3.7		
Education	lower secondary (Hauptschule)	6	2.5		
	intermediate secondary (Realschule)	168	69.4		
	upper secondary (Fach-/Hochschulreife)	55	22.7		
	other	4	1.7		
	NA	9	3.7		
Prior work experience	yes	114	47.1		
	no	115	47.5		
	NA	13	5.4		
Family members working in the care sector	yes	154	63.6		
	no	72	29.8		
	NA	16	6.6		
Residential area	urban	54	22.3		
	suburban	126	52.1		
	rural	44	18.2		
	NA	18	7.4		
Total daily commute time to training workplace (min)		195		35.3	28.3
Total daily commute time to vocational school (min)		195		34.8	27.6

### Questionnaire development

The instrument drew on established survey-design principles [21, 36, 43], on the mixed-method EPAT approach for capturing practical and motivational aspects of healthcare career orientation [7], and on nursing-specific instruments and evidence on motivational drivers, gender- and life-stage differences, intrinsic versus extrinsic motivation, and training-related barriers [3, 5, 19, 22, 27, 51, 52]. Some items were adopted or adapted from earlier work, while others were newly developed to close content gaps identified during the preparatory phase. The final instrument was implemented in the Tivian EFS (Discover XI) software [48] and made available in both online and printed paper-pencil formats.

The item pool was theoretically grounded (cf. Section “Theoretical grounding”), with constructs mapped onto survey sections as follows. The seven motivational items analysed in Sections “Motivational influences on career choice” operationalise the SDT distinction between autonomous regulation (personal interest, prior internship, proximity to home), controlled regulation (media, career counselling, parents/relatives), and instrumental regulation (income prospects). The transport-relevance, commute-stress, and family-care items analysed in Section “Internship experience and geographic accessibility” operationalise the contextual-barrier branch of SCCT and, within the push-pull reading, function as push factors salient at the training-choice stage. The twelve school- and company-based training evaluations analysed in Section “Practical experiences and orientation” operationalise SCCT learning experiences that feed self-efficacy and outcome expectations. The two retention items analysed in Sections “Determinants of future retention intentions” — stay-in-occupation and stay-in-sector — operationalise SCCT’s persistence dimension at two granularities and enable the push-pull reading of lateral sector-internal mobility. This construct-to-item mapping was fixed a priori; the factor analyses reported below test, but do not define, this theoretical structure.

Instrument development followed a multi-phase, iterative approach. Item pools and phrasing were directly informed by findings from three structured workshops with groups of 15–20 apprentices, which captured authentic perspectives on job satisfaction, sources of challenge, and improvement needs with particular regard to school-workplace incoherence and the organization of practical placements. The instrument featured primarily closed-ended items with five-point Likert-type response formats, selected for their psychometric balance between sensitivity and interpretability. The questionnaire additionally comprised ten open-ended items inviting apprentices to elaborate on their motivations, training experiences, and perceived barriers. Their systematic analysis requires thematic coding and inter-coder reliability procedures that fall outside the scope of the present paper and will be reported in a dedicated follow-up study; the quantitative analyses reported here rely exclusively on the closed-ended items. Sociodemographic variables were deliberately focused on age, gender, educational attainment, prior professional experience, living environment (rural vs. urban), and mobility barriers, chosen for their documented relevance in previous studies. The final questionnaire is provided in the Appendix.

We pretested the questionnaire in cognitive interviews with five apprentices, focusing on clarity, item comprehension, and phrasing adequacy. Feedback from this phase resulted in minor refinements to question wording and explanatory instructions. Further validation was sought through consultative feedback from school leadership and area supervisors, ensuring coverage and relevance from an institutional perspective. Internal consistency (Cronbach’s α) for the multi-item scales used in the analyses is reported in the Appendix (Table S3).

## Results

### Motivational influences on career choice

From a self-determination perspective, as introduced in Section “Theoretical grounding”, autonomous motives are expected to be more closely linked to stable and self-concordant career choice than controlled motives. Specifically, in this context we analyse responses to the survey question: “How important were the following aspects when you chose your current apprenticeship?” Participants rated seven potential drivers on a five-point Likert scale (1 = “very important” to 5 = “completely unimportant”): media, career counselling, internship experience, parents/relatives, income prospects, proximity to home, and personal interest. All analyses were conducted using Stata 18 [45].

All response distributions in this paper are visualised as diverging stacked bar charts. In these charts, response categories are displayed symmetrically around a central axis representing the scale midpoint. Positive responses (e.g., “very important”, “very good”) extend to the right; negative responses extend to the left. Darker shades indicate more extreme responses within each direction. The neutral midpoint category is split equally across both sides. Bar lengths are proportional to the percentage of respondents selecting each category, allowing both the overall response tendency and the degree of polarisation to be read directly from the figure.

**Fig. 1:**
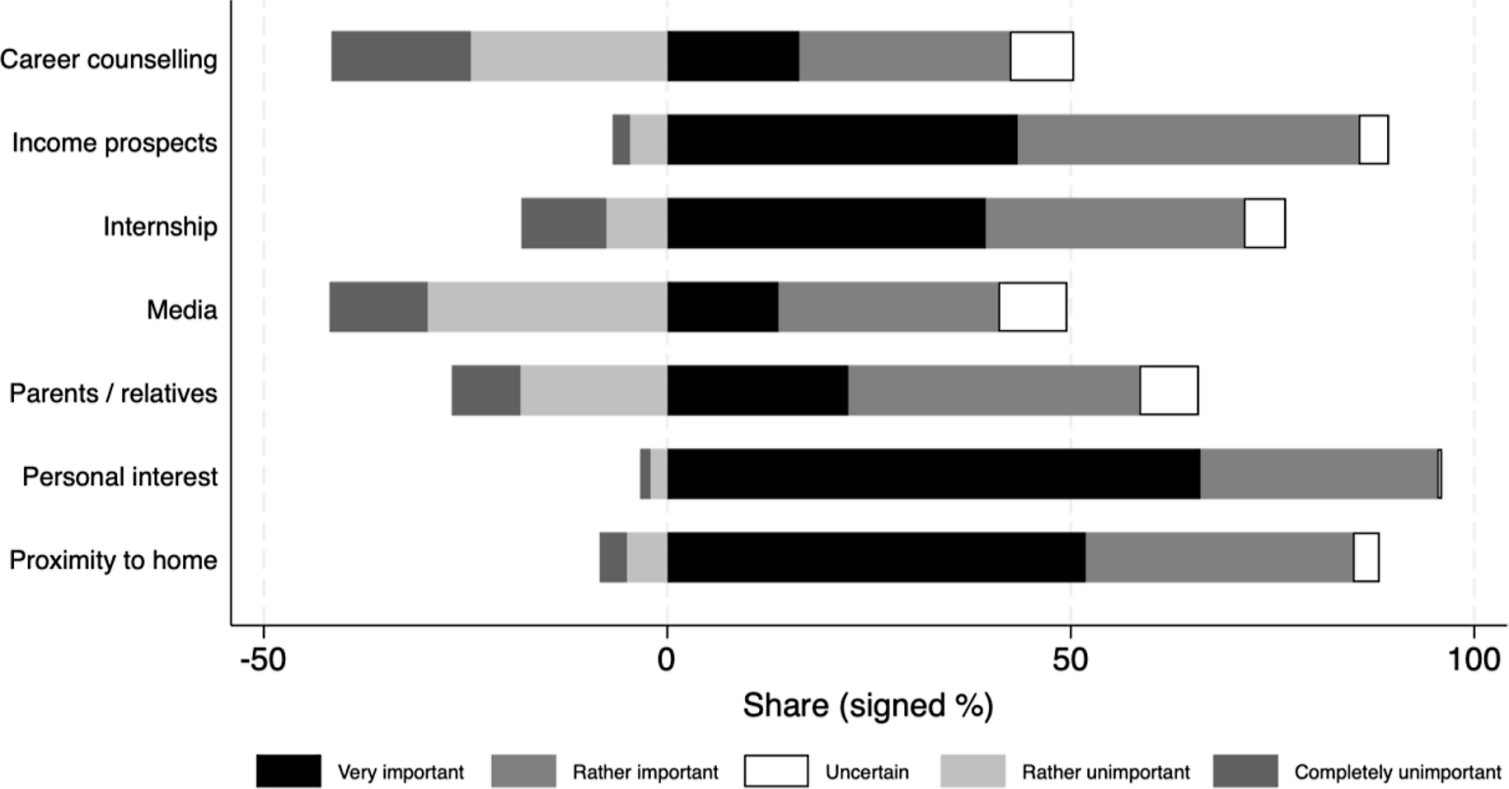
Motivation for choosing the apprenticeship. Diverging stacked bar chart of response distributions across the seven motivational items (1 = very important, 5 = completely unimportant); missing values excluded

Figure 1 displays the response distribution for each factor. We tested whether apprentices attach significantly different importance to these factors using a Friedman test on complete cases (*N* = 233). The omnibus test was highly significant, χ²(6) = 423.50, *p* < 0.001, with Kendall’s W = 0.26 indicating a small to moderate level of agreement in the rank ordering across respondents. Post hoc pairwise comparisons via Wilcoxon signed-rank tests with Holm correction (21 comparisons, family-wise α = 0.05) revealed a hierarchy of five tiers. Personal interest (mean = 1.44) was rated significantly more important than any other factor (all p_Holm < 0.001, *r* = 0.26 to 0.79), followed by proximity to home and income prospects (means 1.74 and 1.79, not significantly different from each other, p_Holm = 0.37), then prior internship (mean = 2.19), parental advice (mean = 2.56), and finally the two traditional information channels. Media exposure and career counselling were statistically indistinguishable (means 3.01 and 3.03, p_Holm = 0.97) and rated significantly less important than all other factors (p_Holm ≤ 0.001). Parental advice thus forms a separate intermediate tier, distinct from media and career counselling (both p_Holm < 0.001).

To explore whether the ranked items reflect a latent dimensional structure that could inform typology-based recruitment, we ran an exploratory factor analysis on the seven five-point items using a polychoric correlation matrix (*N* = 233). Sampling adequacy was acceptable (KMO = 0.71) and Bartlett’s test of sphericity was significant, χ²(21) = 287.70, *p* < 0.001. The factor-retention decision required reconciling several conventional criteria that diverge for these data. The Kaiser criterion applied to either the polychoric or the Pearson correlation matrix retains three factors (polychoric eigenvalues 2.56, 1.14, 1.01; Pearson 2.30, 1.13, 1.02), the third one only marginally above unity. Horn’s parallel analysis (1,000 replications) yielded divergent recommendations depending on the extraction method: the factor-extraction variant retains three factors, whereas the principal-components variant retains only one. We therefore did not treat parallel analysis as a single decisive criterion. Instead, the choice of two factors was made on substantive grounds: the three-factor alternative produces a singleton factor loading exclusively on parental advice, which is not substantively identified [8, 14], while the one-component PCA solution collapses the theoretically meaningful distinction between autonomous and controlled drivers (cf. Section “Theoretical grounding”) into a single dimension. The two-factor solution we retain is consistent with the SDT-grounded a priori construct mapping and with the dominance pattern of the eigenvalues, where the gap between the second and the third is negligible (1.13 → 1.02 in the Pearson sequence) while the gap between the first and the second is pronounced (2.30 → 1.13). The full eigenvalue sequence and parallel-analysis output are reported in Appendix Table S2. We apply an orthogonal Varimax rotation.

Together, the two orthogonal factors extracted by iterated principal-factor analysis on the polychoric correlation matrix explain 36.1% of the total item variance. While this is below the conventional 50% threshold often cited for component-based analyses, the figure is within the range acceptable for common-factor analysis of short ordinal scales [32, 34]. All uniqueness values remain below 0.80, and after Varimax rotation every item loads above 0.41 on its primary factor. The rotated pattern matrix is readily interpretable. Factor 1 “Institutional Guidance” loads saliently on career counselling (0.92), prior internship (0.55) and media exposure (0.48). Factor 2 “Personal & Pragmatic Considerations” loads on personal interest (0.54), income prospects (0.46), parental advice (0.45) and proximity to home (0.42).

Internal consistency of these short ordinal scales is moderate: standardised Cronbach’s α = 0.63 (ordinal α = 0.69) for Factor 1 and α = 0.54 (ordinal α = 0.59) for Factor 2; full results are reported in Appendix Table S3. These values fall below the conventional 0.70 threshold but are expected for ordinal scales of three to four items, where α is structurally attenuated by item count and by the ordinal level of measurement [41, 47]. Crucially, the two factors are used here not as composite scores for individual prediction but as latent-structure descriptors that inform the cluster analysis below; their interpretation is anchored a priori in SDT (cf. Section “Theoretical grounding”) rather than derived purely from data. By contrast, the school- and company-based training-quality scales used in Sections “Practical experiences and orientation” and “Determinants of future retention intentions” achieve good internal consistency (α = 0.82 and 0.86, respectively; Appendix Table S3).

Building on the factor scores, we performed a k-means cluster analysis to identify apprentice profiles along the two latent dimensions. Cluster selection was based on two complementary criteria: the Calinski–Harabasz (CH) pseudo-F and the mean silhouette width [23], with cluster sizes inspected for interpretative stability. Table 2 reports both criteria for k = 2 to k = 10. The two-cluster solution maximises the mean silhouette width (0.46) and yields a near-maximal local CH value (212.75); it also produces balanced and interpretable groups (N1 = 105, N2 = 128). The global CH maximum at k = 9 (CH = 219.55) was rejected as it yields an average cluster size of only 26 respondents, characteristic of over-segmentation in moderate samples.

**Table 2:** Cluster diagnostics for k-means solutions on Factor 1 / Factor 2 scores

k	Calinski-Harabasz	Mean silhouette
2	212.75	0.455
3	206.80	0.447
4	187.33	0.354
5	174.07	0.344
6	173.81	0.378
7	167.98	0.361
8	187.91	0.388
9	219.55	0.424
10	209.95	0.420

Note: Calinski-Harabasz pseudo-F (higher = better separation, original Calinski & Harabasz 1974) and mean silhouette width (Kaufman & Rousseeuw 1990; > 0.50 reasonable, > 0.25 weak but useful) computed on Euclidean distances over the two retained Varimax-rotated factor scores. *N* = 233 complete cases

Interpretation: the highest mean silhouette and a local CH peak both occur at k = 2; at k = 9 the CH index is marginally higher (by 3 %) but the silhouette is lower, and we therefore select k = 2 on the basis of silhouette dominance and parsimony.

The resulting two-cluster solution maps directly onto the two extracted factors. Cluster 1 “Personal-Pragmatic apprentices” (*N* = 105) shows elevated scores on Factor 2 and low to negative scores on Factor (1) Members’ career decisions are shaped primarily by personal interest, income expectations, commuting distance and family influence, with external institutional guidance playing a subordinate role. Cluster 2 “Institutionally Guided apprentices” (*N* = 128) shows the mirror-image profile, with high scores on Factor 1 and low scores on Factor (2) Decisions here rely more strongly on formal information sources (career counselling, internships and media exposure) while personal and contextual constraints carry less weight.

Beyond the descriptive ranking of individual motives, the two-cluster solution adds a person-centred layer to the analysis. It indicates that apprentices differ not only in the strength of single motives, but also in the overall configuration of their career-choice orientation: one group is characterised by a more self-directed and pragmatically grounded profile, whereas the other relies more strongly on institutional guidance and external information sources. The typology is presented descriptively here and is not used as an independent variable in the subsequent analyses.

### Internship experience and geographic accessibility

According to Section “Theoretical grounding”, entry into training is not determined by motivation alone; it also depends on whether applicants perceive the pathway as accessible, visible, and feasible. Two aspects are examined in this context: (i) prior internship experience and (ii) the relevance of geographic accessibility. The survey captured these aspects through the following items:

*Did you complete an internship as part of your career orientation?”* (yes/no; the item refers to any career-orientation internship and is not restricted to the healthcare sector), and

*“To what extent did the accessibility of the training institution (transport connections) influence your decision for an apprenticeship or internship?”* with a five-point response scale (1 = very important to 5 = not important at all).

Because this subsection involves twelve exploratory sub-group comparisons in total, we control the false-discovery rate at q = 0.05 using the Benjamini–Hochberg procedure [2] and report adjusted p-values (p_BH) alongside the unadjusted values where they affect inference.

Table 3 reports the share of apprentices who completed an internship prior to entering their training program, disaggregated by demographic subgroups. For each cross-tabulation we report the Pearson χ²-test together with Cramér’s V as an effect-size measure. The propensity to undertake an internship is associated with age (χ²(4) = 11.13, *p* = 0.025, V = 0.22), although this association does not survive multiplicity correction (p_BH = 0.08). No meaningful associations emerge for gender (χ²(1) = 0.26, *p* = 0.61, V = 0.03), school attainment (χ²(4) = 1.54, *p* = 0.82, V = 0.08), or previous work experience (χ²(1) = 0.12, *p* = 0.73, V = 0.02), with Cramér’s V below the conventional small-effect threshold of 0.10 throughout. For family members already working in healthcare, the association does not reach conventional significance (χ²(1) = 3.43, *p* = 0.06, V = 0.12, p_BH = 0.13).

**Table 3:** Internship participation by demographic and contextual characteristics

Variable	*n*	chi-square	df	*p*	Cramer’s V	p_BH
Age (5 categories)	224	11.13	4	0.025	0.223	0.076
Gender	233	0.26	1	0.610	0.033	0.732
School attainment	233	1.54	4	0.819	0.081	0.819
Prior work experience	229	0.12	1	0.732	0.023	0.799
Family in healthcare	226	3.43	1	0.064	0.123	0.128

Note: Pearson chi-square tests with Cramer’s V as effect size (Cohen’s guideline: V = 0.10 small, 0.30 medium, 0.50 large). p_BH = Benjamini-Hochberg adjusted p-value, pooled across all 12 sub-group tests reported in Section “Internship experience and geographic accessibility”

Sample restricted to apprentices with non-missing data on the respective demographic variable; family in healthcare and prior work experience are dichotomous (yes/no)

**Fig. 2:**
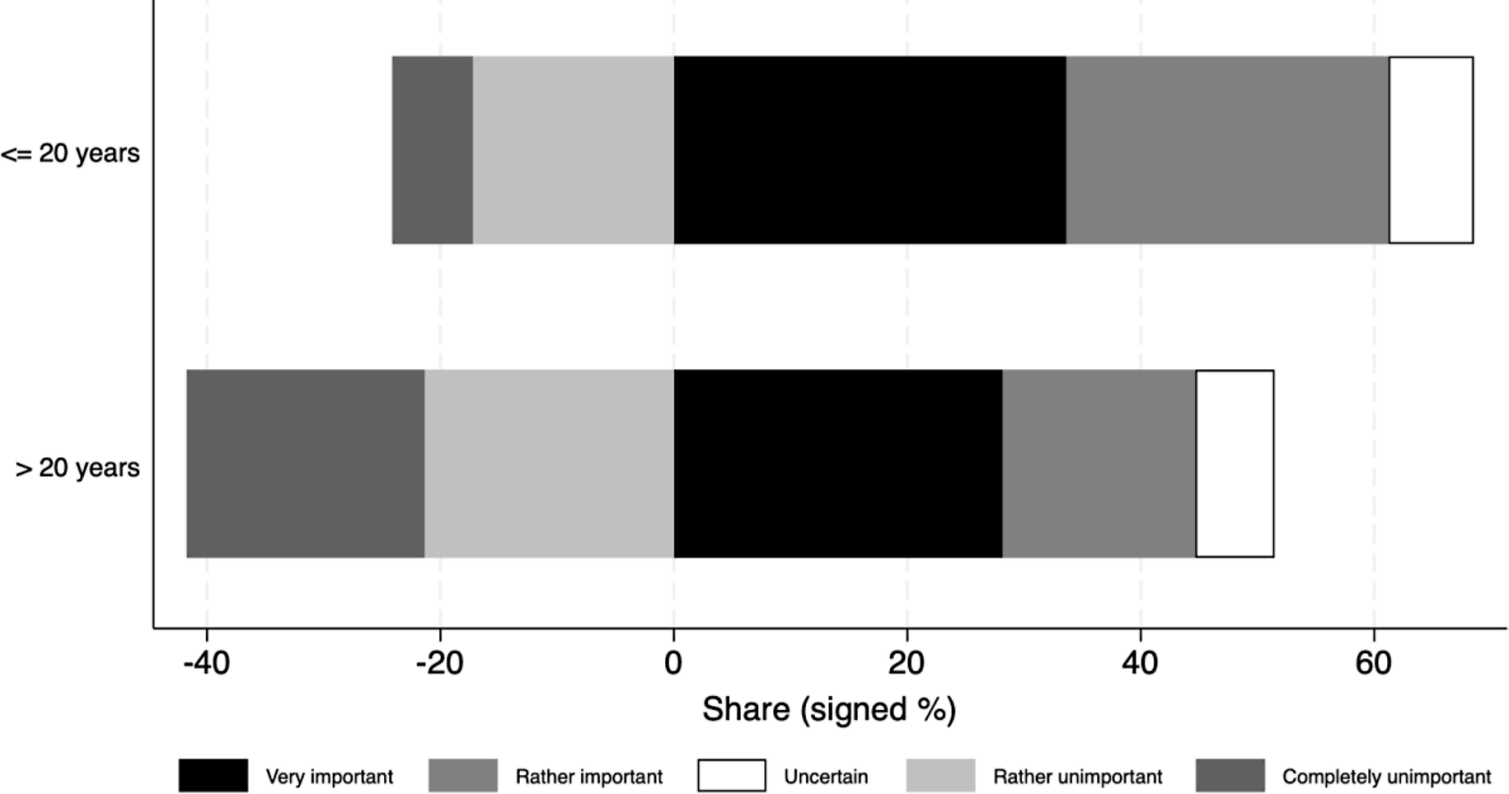
Relevance of transport connections by age group. Diverging stacked bar chart of responses to the transport-relevance item (1 = very important, 5 = not important at all), stratified by age group (≤ 20 years vs. > 20 years)

Another factor frequently discussed in the literature concerns the importance of transport connections when choosing an apprenticeship or internship site. Because the transport-relevance item is measured on a five-point ordinal scale, we analyse it using rank-based non-parametric tests: Mann–Whitney U tests for binary groupings with the rank-biserial correlation r = Z/√N as an effect-size measure [24], and Kruskal–Wallis H tests for multi-category groupings with η²_H = (H − k + 1)/(N − k) as an effect-size measure [49]. Figure 2 displays the distribution of ratings as diverging stacked bars by age group (≤ 20 vs. > 20 years).

Consistent with expectations, there is a significant association between age group and the importance of transport connections: apprentices aged ≤ 20 years rate accessibility as more important than those aged > 20 years (z = − 2.52, *p* = 0.01, *r* = − 0.17, p_BH = 0.047, *n* = 219). The Kruskal–Wallis test across the full five-category age variable yields the same directional pattern but narrowly misses the unadjusted 5%-threshold (H(4) = 9.38, *p* = 0.052, η²_H = 0.03, p_BH = 0.13). In contrast to the impression suggested by a χ²-test on the ordinal item, the Mann–Whitney U test finds no evidence of a systematic gender difference in perceived transport relevance (z = 1.03, *p* = 0.30, *r* = 0.07, *n* = 228).

Beyond age and gender, Table 4 (Kruskal–Wallis section) further disaggregates the relevance of transport connections by additional demographic characteristics. No substantive association emerges with educational attainment (H(4) = 7.49, *p* = 0.11, η²_H = 0.02, p_BH = 0.19). Somewhat unexpectedly, there is a significant relationship with having family members employed in the healthcare sector (z = − 2.77, *p* = 0.01, *r* = − 0.19, *n* = 221, p_BH = 0.03): apprentices from such families assign higher importance to accessibility. One plausible explanation is that family exposure may sensitize individuals to the challenges posed by transport connections when entering and sustaining a career in healthcare. By contrast, no significant differences are observed by residential location (urban, small town, rural; H(2) = 1.95, *p* = 0.38, η²_H ≈ 0, p_BH = 0.50).

Finally, Table 4 examines whether prior internship experience influences perceptions of transport relevance. A Mann–Whitney U test confirms a significant association (z = − 3.74, *p* < 0.001, *r* = − 0.25, *n* = 233, p_BH < 0.01): apprentices who completed an internship before entering their training are more likely to rate accessibility as “very important”. This finding suggests that practical exposure may sharpen awareness of logistical barriers, much as family exposure does. After Benjamini–Hochberg correction across the twelve sub-group comparisons in this subsection, three associations remain statistically supported: prior internship experience (p_BH < 0.01, *r* = − 0.25), family employment in healthcare (p_BH = 0.03, *r* = − 0.19), and age group (p_BH = 0.047, *r* = − 0.17), all of small to small-to-moderate magnitude and all pointing towards higher perceived relevance of transport accessibility.

**Table 4:** Relevance of transport connections by demographic and contextual characteristics

Mann-Whitney U tests (binary groupings)						
**Variable**	**n**	**z**	**p**	**r**	**p_BH**	
Age group (<= 20 vs. > 20 years)	219	-2.52	0.012	-0.170	0.047	
Gender (male vs. female)	228	1.03	0.302	0.068	0.453	
Family in healthcare (yes vs. no)	221	-2.77	0.006	-0.186	0.034	
Prior internship (yes vs. no)	233	-3.74	0.000	-0.245	0.002	
***Kruskal-Wallis H tests (multi-category groupings)***						
**Variable**	**n**	**H**	**df**	**p**	**eta^2_H**	**p_BH**
Age (5 categories)	219	9.38	4	0.052	0.025	0.126
School attainment	228	7.49	4	0.112	0.016	0.192
Residential environment	219	1.95	2	0.377	0.000	0.503

Note: Five-point ordinal outcome (1 = very important, 5 = completely unimportant). Mann-Whitney U for binary groupings with rank-biserial r = z / sqrt(N) (Kerby 2014); Kruskal-Wallis H for multi-category groupings with eta^2_H = (H - k + 1) / (N - k) (Tomczak & Tomczak 2014). p_BH = Benjamini-Hochberg adjusted p-value, pooled across all 12 sub-group tests reported in Section “Internship experience and geographic accessibility” Effect-size guides: |r| = 0.10 small, 0.30 medium, 0.50 large; eta^2 = 0.01 small, 0.06 medium, 0.14 large

Sample restricted to respondents with non-missing data on transport relevance and the respective demographic variable. Gender restricted to male/female respondents; the small ‘diverse’ category is excluded from MWU

### Practical experiences and orientation

Within SCCT, learning experiences influence self-efficacy beliefs and outcome expectations, which in turn affect both willingness to remain in training and the longer-term stability of occupational perceptions. The survey allows us to draw on two items related to the following survey question:

*Looking at your current training: What works well*,* and what could be improved?*

Ratings were provided on a five-point Likert scale for distinct dimensions. For the school-based part of training, the assessed dimensions included course organisation, didactic guidance, curricular content, school instructors, classroom climate, and relationships with classmates. For the company-based part of training, respondents evaluated workflow in the company, on-the-job instruction, practical content, company trainers, climate among trainees, and relationships with co-workers (see the Appendix for exact wording).

**Fig. 3:**
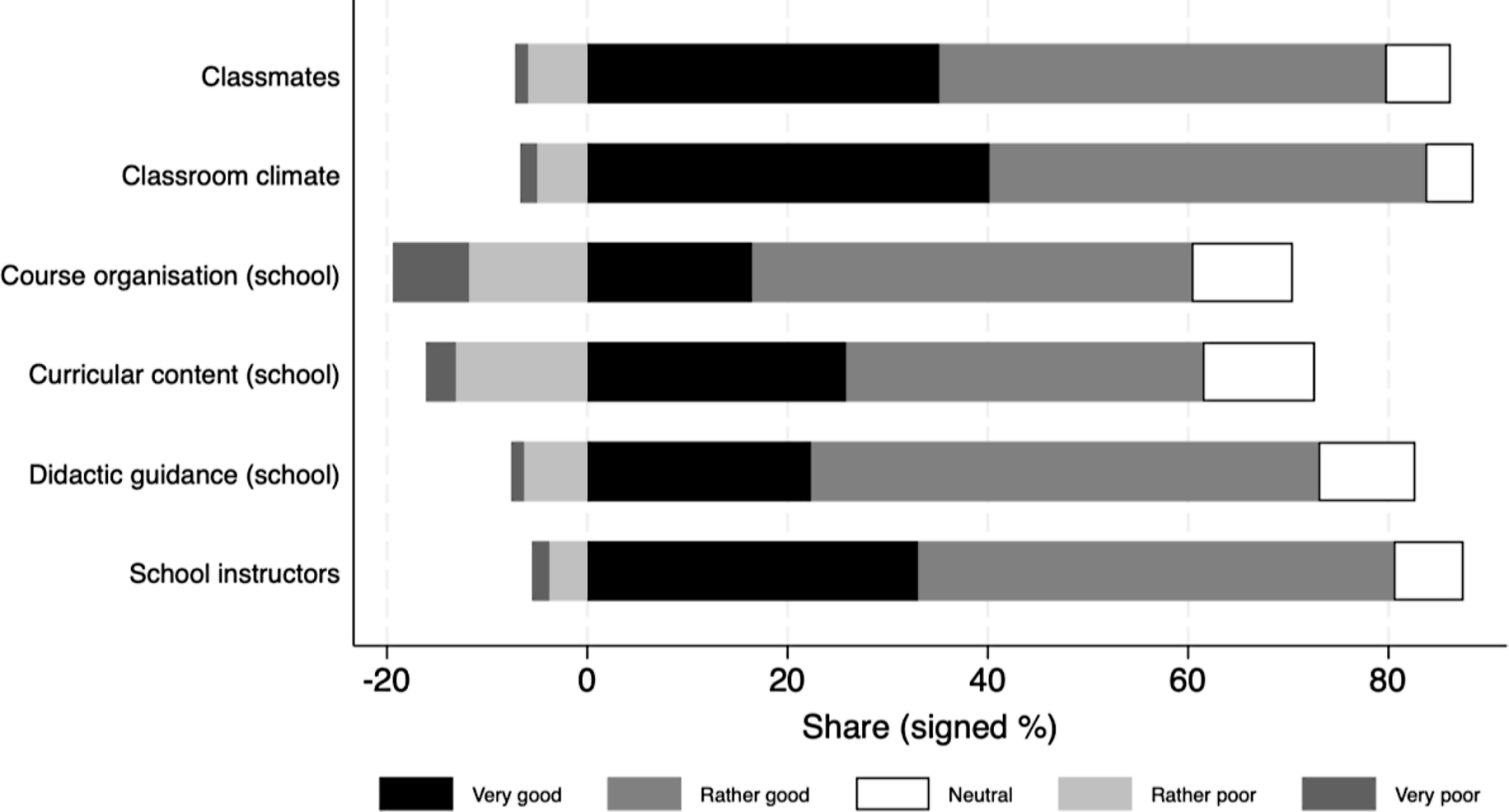
School-related training ratings. Diverging stacked bar chart of response distributions across the six school-related training-quality items (1 = very good, 5 = very poor); missing values excluded

**Fig. 4:**
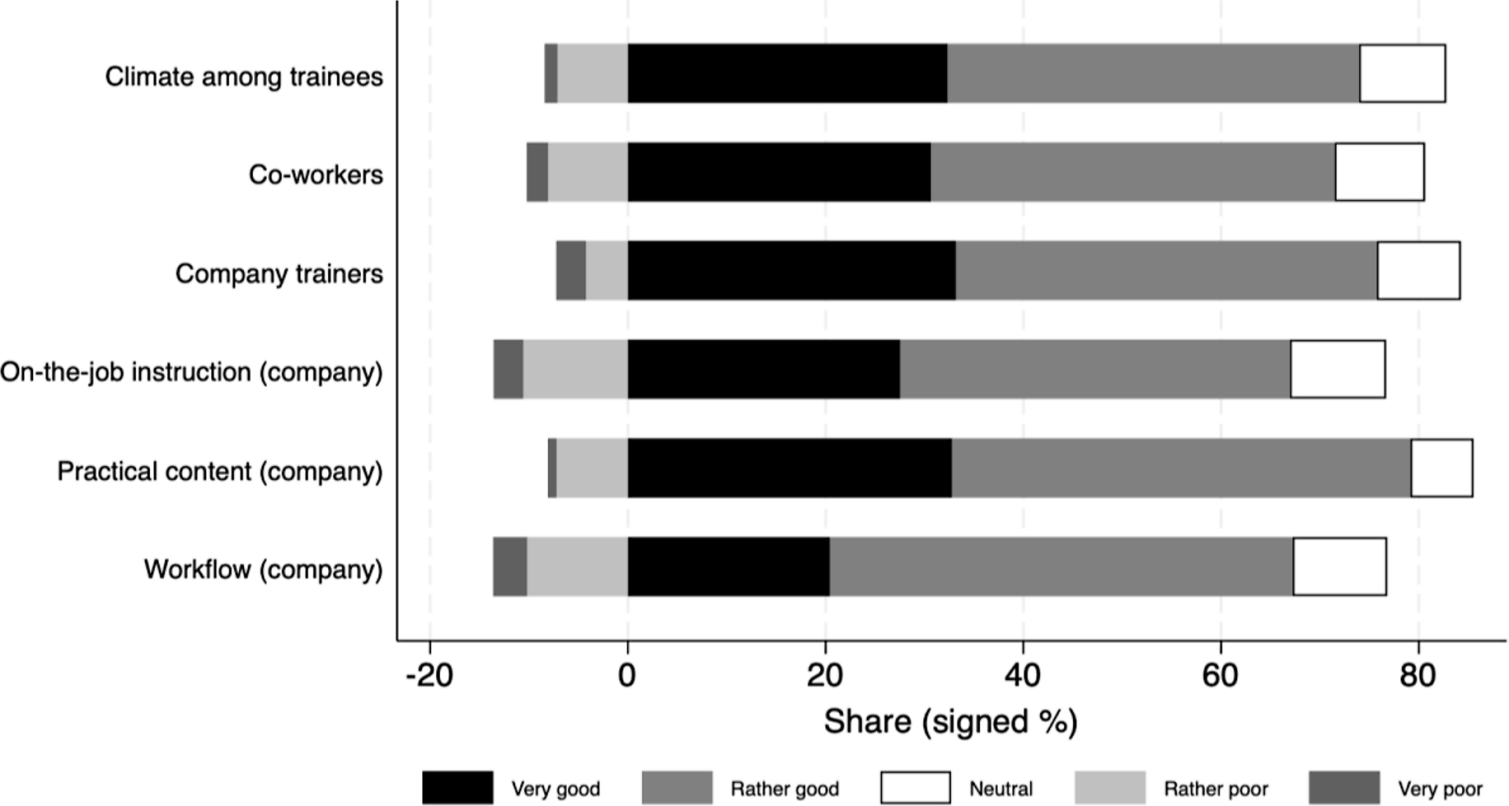
Company-related training ratings. Diverging stacked bar chart of response distributions across the six company-related training-quality items (1 = very good, 5 = very poor); missing values excluded

Figures 3 and 4 display the response distributions for these dimensions as diverging stacked bar charts (consistent with Fig. 1 in Sections “Motivational influences on career choice”), separately for the school and workplace components of the dual training system.

Figures 3 and 4 show an overall positive assessment of both the school-based and company-based components of training. Across all dimensions, between roughly 60% and 85% of responses fall into the “very good” or “rather good” categories, contradicting to some extent the common public perception of healthcare training as a predominantly “difficult” or negative experience.

To formally test whether evaluations differ across the six domains within each training context, we conducted separate Friedman tests for the school-related and company-related items. For the school-related domains, the test indicates pronounced overall differences (χ²(5) = 115.86, *p* < 0.001; Kendall’s W = 0.14, reflecting small-to-moderate consensus). For the company-related domains, the omnibus test is also highly significant (χ²(5) = 46.44, *p* < 0.001; W = 0.06, indicating little rank-order differentiation); we therefore complement the omnibus with targeted pairwise contrasts. To identify which specific domains differ, we supplement the omnibus tests with pairwise Wilcoxon signed-rank tests within each context (15 comparisons each), applying Holm’s step-down correction to control the family-wise error rate at α = 0.05 (full results in Appendix Table S4), and report r = |z|/√N as an effect-size measure [15].

Within the school-based context, 12 of 15 pairwise comparisons are Holm-significant. Course organisation receives the least favourable ratings and differs from instructors, classroom climate, and classmates with large effect sizes (*r* = 0.47–0.54). Curricular content receives the next-least favourable ratings and differs from all five other domains (*r* = 0.17–0.34). No significant differences emerge among school instructors, classroom climate, and classmates (all p_Holm > 0.28), which form the most favourably rated tier.

Within the company-based context, 6 of 15 pairwise comparisons are Holm-significant. Workflow/organisation and on-the-job instruction are rated significantly lower than the remaining four domains in 6 of 8 such pairs (*r* = 0.23–0.37; all p_Holm ≤ 0.005), with on-the-job instruction vs. climate among trainees and vs. co-workers not reaching significance after correction (p_Holm = 0.12 and 0.14). No significant differences emerge among practical content, company trainers, climate, and co-workers (all pairwise p_Holm = 1.00; *r* ≤ 0.09).

We next examined whether apprentices differentiate between identical domains when assessed for school versus company settings. Paired Wilcoxon signed-rank tests (n between 234 and 238 per pair) indicate that workflow/organisation and practical content receive significantly more favourable ratings in the company than in school (z = 2.46, *p* = 0.01, *r* = 0.16; z = 4.10, *p* < 0.001, *r* = 0.27, respectively). Conversely, the social climate among trainees and co-workers is judged slightly less favourably than the corresponding classroom climate and relationships with classmates (z = − 2.50, *p* = 0.01, *r* = 0.16; z = − 2.12, *p* = 0.03, *r* = 0.14). No reliable differences were found for on-the-job instruction vs. didactic guidance (z = − 0.86, *p* = 0.39, *r* = 0.06) or for company trainers vs. school instructors (z = − 0.87, *p* = 0.38, *r* = 0.06).

To explore whether perceptions of school-based training quality are linked to apprentices’ future career intentions, we computed Spearman rank correlations between each of the six school-based rating items and the two retention outcomes (listwise). Career intention was measured with the item:

*“Do you think that the occupation you are currently training for will still be suitable for you in the future?”* (five-point scale assessing the likelihood of remaining with the current employer or in the profession).

Because this procedure entails twelve simultaneous tests, we apply a Benjamini–Hochberg false-discovery-rate correction at q = 0.05. After correction, four of six correlations remain significant for retention in the occupation and four of six for retention in the sector, with curricular content showing the strongest association in both cases (ρ = 0.26 and 0.25, respectively; both p_BH ≤ 0.002). For retention in the sector, school instructors and classroom climate fall just short of the threshold (p_BH = 0.053 in both). School instructors and classroom climate do not survive multiplicity correction in either outcome. Full results are reported in Appendix Table S5. Although effect sizes are small throughout, the consistent positive direction — robust to multiplicity correction — suggests that higher satisfaction with school-related learning conditions is modestly associated with stronger retention intentions in both the occupation and the sector.

Beyond evaluations of training content and learning conditions, we additionally examine commuting distance and accessibility during training as further structural conditions shaping practical experience in rural settings. While Section “Internship experience and geographic accessibility” examined the ex ante importance of transport connections in choosing an apprenticeship, we additionally capture ex post experiences through the item:

*How stressful do you find the commuting distance in connection with your training?*

Responses were recorded on a five-point scale (1 = “very stressful” to 5 = “not stressful at all”) and were provided separately for the school-based and company-based components of the program.

**Fig. 5:**
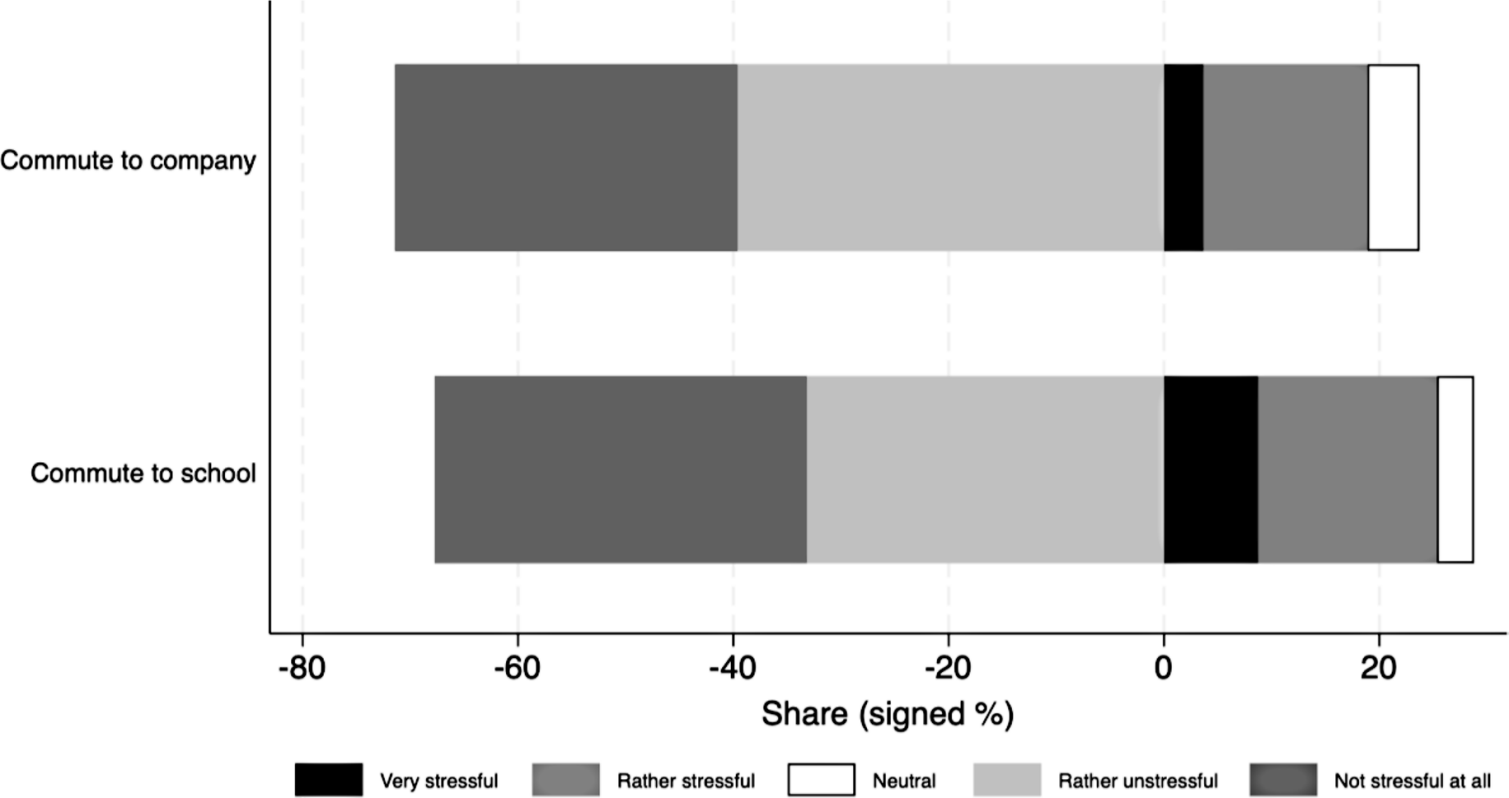
Perceived commute stress. Diverging stacked bar chart of response distributions for commute stress to school and to the training company (1 = very stressful, 5 = not stressful at all); missing values excluded

Figure 5 displays the distribution of responses regarding perceived commuting stress as diverging stacked bars. Contrary to the frequently voiced anecdotal narrative of stressful travel routes in vocational healthcare training, around 70% of apprentices report that commuting is “rather unstressful” or “not stressful at all.” A Wilcoxon signed-rank test comparing the perceived stress for commuting to the workplace versus to the vocational school shows no systematic difference (z = − 0.42, *p* = 0.67, *r* = 0.03, *n* = 216).

In addition to motivational factors, the study also examines changes in apprentices’ perceptions of their chosen occupation after training has begun. Large discrepancies between initial expectations and subsequent experiences can create frustration and increase the risk of dropout or switching to another field. Understanding these changes therefore provides potential entry points for decision-makers to make healthcare training more sustainable. To capture this aspect, respondents were asked:

*Since you made the decision for your current apprenticeship*,* has your view of the profession changed?”* with five response categories ranging from “changed very strongly” to “not changed at all.

To examine whether prior internship experience is associated with changes in apprentices’ perceptions of the chosen occupation, we conducted a Mann–Whitney U test on the ordinal five-point response item, which preserves its rank structure. The test indicates no significant association (z = 1.38, *p* = 0.17, *r* = 0.09, *n* = 237). More generally, the distribution of responses paints a less dramatic picture than is often portrayed in public discourse: for more than 50% of apprentices, perceptions of the occupation have changed only slightly or not at all, while only about 9% report a strong change (note that the direction of change — positive or negative — was not assessed).

These results underline the importance of a more differentiated analysis of factors that may influence changes in occupational perceptions, which is the focus of Sections “Determinants of future retention intentions”.

### Determinants of future retention intentions

Within a push-pull perspective, dissatisfaction with training experiences may translate into different exit pathways: apprentices may not leave healthcare altogether, but may instead look for a different role within the sector [1]. This section therefore examines the factors associated with apprentices’ intentions to remain in their chosen occupation and in the broader healthcare sector. Understanding these drivers is of direct practical relevance for developing strategies to strengthen the long-term workforce base in nursing and related fields. Retention intentions were captured in the survey with the item:

*Do you think that the occupation you are currently training for will still be suitable for you in the future?*

**Fig. 6:**
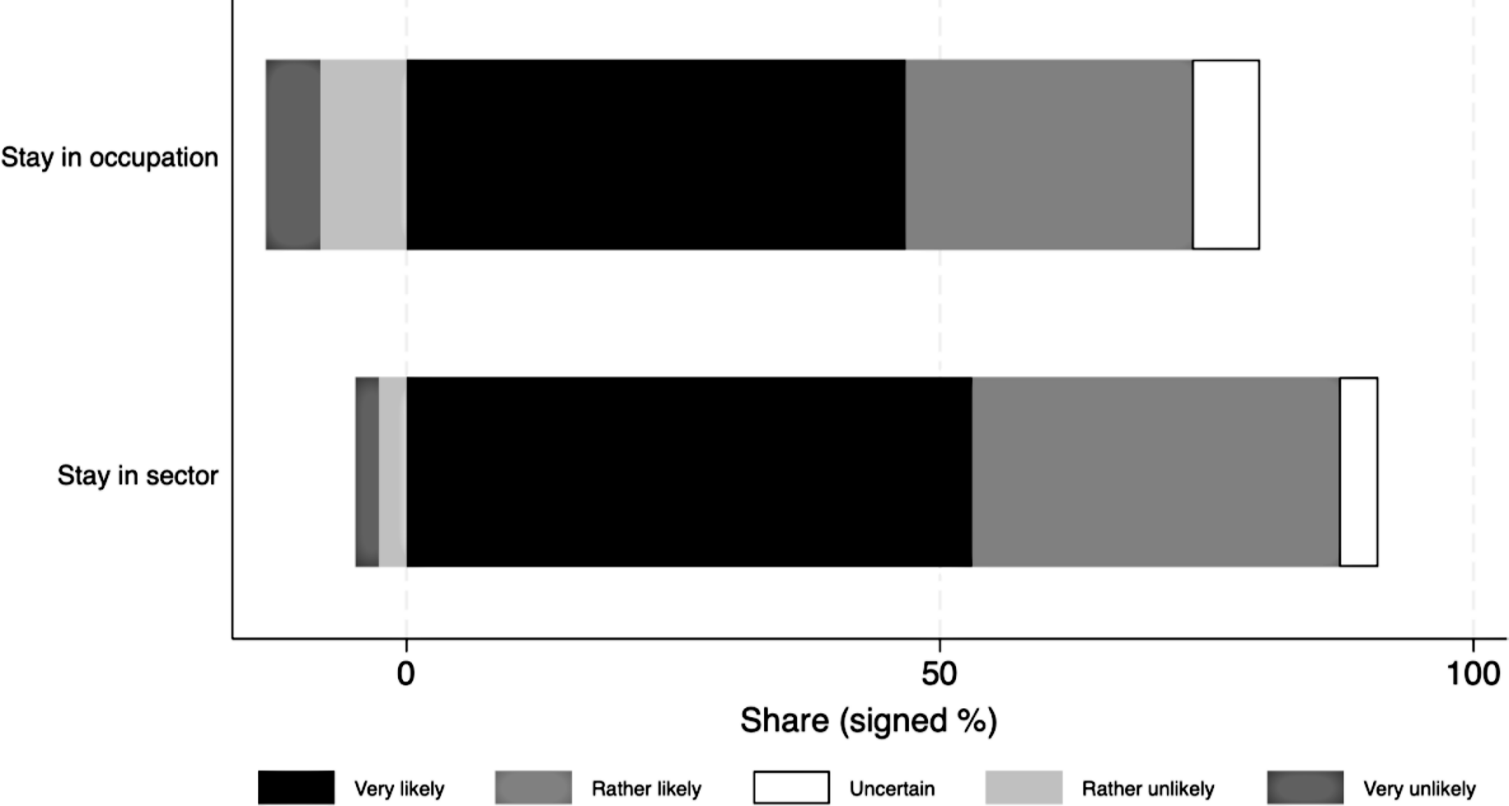
Likelihood of staying in current occupation/sector. Diverging stacked bar chart of response distributions for the two retention items (1 = very likely, 5 = very unlikely); missing values excluded

Respondents indicated their likelihood on a five-point scale ranging from “very likely” to “very unlikely”, separately for (i) remaining in the specific occupation and (ii) remaining in the broader sector. Figure 6 displays the distribution of responses for both items as diverging stacked bars.

The descriptive results — again running counter to a frequently voiced public narrative — draw a surprisingly positive picture of apprentices’ willingness to remain in the healthcare field. Roughly three quarters of respondents (74%) indicate that they are “very likely” or at least “rather likely” to stay in their specific occupation, and 87% indicate this for the broader sector. A Wilcoxon signed-rank test rejects the null hypothesis of identical response distributions (z = 4.14, *p* < 0.001, *r* = 0.27, *n* = 229). Apprentices thus clearly distinguish between remaining in their exact occupation and remaining somewhere within the wider healthcare sector, with a roughly 6-percentage-point higher share opting for “very likely” when asked about the sector than about the occupation. This suggests that switching to a related occupational field within healthcare may be a realistic pathway for some trainees.

To identify factors associated with the probability of remaining in the occupation or sector, we estimated ordinal logistic regression models, which respect the ordinal nature of the five-point outcome scale. As main explanatory variables, we included the twelve rating items from Section “Practical experiences and orientation” that capture satisfaction with the school- and company-based components of training, because these are conceptually well-justified predictors of retention. In addition, socio-economic covariates were incorporated (see Table 4) to account for demographic and contextual factors — such as prior work experience, the existence of prior internships, and the perceived importance of internships for career choice — that may also influence retention intentions. Following the first-round review, we reduced the original specification by excluding two variables for which theoretical or empirical grounds argue against inclusion: “planned relocation”, for which endogeneity with retention intentions cannot be ruled out, and “residential environment”, for which Section “Internship experience and geographic accessibility” provides no evidence of a substantive association with related outcomes. Additionally, the school-attainment variable was collapsed into a binary indicator (lower/intermediate vs. upper secondary), given the very small cell counts in the original five-category coding. Linear regression estimates are reported as a robustness check in Appendix Table S1, together with multicollinearity diagnostics (all VIFs < 2.4) and heteroskedasticity tests. We further note that, owing to listwise deletion across all predictor and outcome variables, the effective sample size for the multivariate regressions ranges from *N* = 172 to *N* = 177; the predictor-to-observation ratio is therefore approximately 1:8, slightly below the conventional 1:10 rule of thumb. We mitigate this by interpreting individual coefficients with appropriate caution and by emphasising patterns that are consistent across the ordinal logistic main models, the OLS robustness checks reported in Appendix Table S1, and the bivariate analyses of Section “Practical experiences and orientation”. The variable “Change in perceptions of the occupation” — measured by the five-point item introduced at the end of Section “Practical experiences and orientation” — was deliberately excluded as an independent variable as it may be considered endogenous to the training process. It is examined separately at the end of this section.

Table 5 presents the regression results for retention intentions in the occupation and sector. Given the coding of the response scales, positive coefficients indicate a higher likelihood of leaving the occupation or the sector. Among the predictors, school-based curricular content stands out as a significant positive factor in both models (β = 0.55, *p* = 0.003 for occupation; β = 0.39, *p* = 0.04 for sector): because these variables are coded in the same way as school grades (see Section “Practical experiences and orientation”), poorer evaluations of curricular content significantly increase the probability of leaving the occupation as well as the sector. This result is intuitively interpretable and highlights a clear leverage point for improving training outcomes in practice. A similar pattern is observed for workflow and organisational processes in the company, which exert a significant positive effect on the likelihood of leaving the occupation (β = 0.47, *p* = 0.02) but not on leaving the sector. For the occupation outcome, age also emerges as a significant predictor (β = −0.49, *p* = 0.004): older apprentices show a stronger intention to remain in their occupation, consistent with the interpretation that later entrants into training have typically made a more deliberate career choice. For the sector outcome, on-the-job instruction (β = 0.45, *p* = 0.04) and co-workers (β = 0.52, *p* = 0.03) emerge as additional significant factors, suggesting that the day-to-day company environment plays a stronger role for sector retention than for occupational retention.

**Table 5:** Ordinal logistic regression results for retention intentions in the occupation and sector

Predictor	beta_occ	SE_occ	p_occ	beta_sec	SE_sec	p_sec
Course organisation (school)	-0.21	0.21	0.317	0.02	0.21	0.933
Workflow (company)	0.47	0.21	0.024	0.05	0.22	0.836
Didactic guidance (school)	0.31	0.24	0.195	0.00	0.25	0.998
On-the-job instruction (company)	0.26	0.21	0.217	0.45	0.22	0.044
Curricular content (school)	0.55	0.19	0.003	0.39	0.19	0.043
Practical content (company)	-0.37	0.26	0.158	-0.05	0.28	0.861
School instructors	-0.32	0.24	0.186	0.10	0.25	0.688
Company trainers	0.33	0.20	0.092	-0.09	0.23	0.703
Classroom climate	0.07	0.21	0.720	-0.10	0.22	0.664
Climate among trainees	-0.18	0.21	0.387	-0.34	0.22	0.114
Classmates	0.14	0.23	0.530	0.30	0.25	0.240
Co-workers	-0.14	0.23	0.548	0.52	0.23	0.026
Age group	-0.49	0.17	0.004	0.01	0.17	0.957
Gender (female)	-0.19	0.33	0.560	0.10	0.35	0.778
Family in healthcare (no)	0.25	0.34	0.468	0.31	0.36	0.382
Commute stress (company)	-0.21	0.15	0.146	-0.17	0.16	0.286
Commute stress (school)	0.07	0.13	0.617	0.11	0.14	0.416
School attainment (upper secondary)	0.52	0.37	0.161	0.07	0.40	0.867
Prior work experience (no)	0.12	0.32	0.703	-0.42	0.34	0.224
Prior internship (no)	-0.34	0.37	0.358	-0.66	0.41	0.105
Internship as motivation	0.28	0.14	0.048	0.21	0.15	0.169
Transport relevance (ex ante)	0.00	0.12	0.981	0.03	0.13	0.832
***Model statistics***						
N	176			172		
McFadden pseudo-R^2	0.114			0.105		
LR chi-square	54.83			38.31		
df	22			22		
p (LR test)	0.000			0.017		

Note: Coefficients on the log-odds scale; positive coefficients indicate a higher likelihood of leaving (i.e., higher response category on the 1-5 scale, where 5 = very unlikely to stay). Reference categories: female (gender), no family in healthcare, lower/intermediate secondary (school attainment), no prior work experience, no prior internship. Predictors: 12 training-quality items + 10 demographic/contextual covariates

OLS robustness coefficients (with VIF and heteroskedasticity diagnostics) are reported in Appendix Table S1

Finally, we examine which factors may be associated with changes in apprentices’ perceptions of their chosen occupation since deciding to enter training, cf. Table 6. For conceptual consistency, the same set of independent variables was used as in the previous regression model, and the model was again estimated using ordinal logistic regression.

The dependent variable, as shown in the questionnaire in the Appendix, is coded such that higher values correspond to smaller changes in occupational perceptions. Accordingly, positive regression coefficients indicate that a given predictor is associated with more stable or consistent views of the occupation over time.

The regression results can be interpreted as follows: negative and significant coefficients for company workflow (β = −0.69, *p* = 0.001), school-based didactic guidance (β = −0.54, *p* = 0.02), and school-based curricular content (β = −0.54, *p* = 0.003) indicate that dissatisfaction in these areas is associated with stronger changes in occupational perceptions over time. This pattern aligns well with intuitive expectations and practical experience: unsatisfactory learning conditions — whether at school or in the company — tend to alter apprentices’ views of the profession more strongly. We further note that, in the bivariate analysis of Section “Practical experiences and orientation”, prior internship experience is not associated with change in career image (Mann–Whitney U: z = 1.38, *p* = 0.17, *n* = 237). In the present multivariate ologit specification, the corresponding coefficient is β = −0.66, *p* = 0.057 — i.e. just above the 5% significance threshold and substantially less robust than would be expected for a substantively meaningful effect. The earlier internship-paradox interpretation in the original submission, which had been based on a more strongly significant OLS coefficient, is therefore not supported by the present, methodologically more appropriate specification.

**Table 6:** Ordinal logistic regression results for change in apprentices’ perceptions of the chosen occupation

Predictor	beta	SE	*p*
Course organisation (school)	0.17	0.19	0.379
Workflow (company)	-0.69	0.21	0.001
Didactic guidance (school)	-0.54	0.23	0.018
On-the-job instruction (company)	-0.03	0.21	0.896
Curricular content (school)	-0.54	0.18	0.003
Practical content (company)	0.42	0.25	0.094
School instructors	-0.05	0.23	0.826
Company trainers	0.03	0.21	0.893
Classroom climate	-0.24	0.20	0.222
Climate among trainees	0.13	0.20	0.510
Classmates	-0.07	0.22	0.736
Co-workers	-0.26	0.22	0.238
Age group	0.00	0.15	0.999
Gender (female)	-0.27	0.31	0.389
Family in healthcare (no)	0.05	0.32	0.876
Commute stress (company)	0.04	0.14	0.800
Commute stress (school)	0.12	0.13	0.334
School attainment (upper secondary)	0.26	0.35	0.452
Prior work experience (no)	0.39	0.31	0.204
Prior internship (no)	-0.66	0.35	0.057
Internship as motivation	0.18	0.13	0.169
Transport relevance (ex ante)	-0.18	0.12	0.128
***Model statistics***			
N	177		
McFadden pseudo-R^2	0.122		
LR chi-square	63.00		
df	22		
p (LR test)	0.000		

Note: Higher response values correspond to smaller change; positive coefficients therefore indicate more stable perceptions. Reference categories as in Table 5. The bivariate Mann-Whitney U test of career-image change by prior internship is non-significant Section “Practical experiences and orientation” the regression coefficient is therefore best interpreted in the context of suppression effects in a multivariate specification with correlated training-quality predictors

OLS robustness coefficients are reported in Appendix Table S1

These results underline the role of concrete training-environment conditions in shaping how apprentices’ views of their chosen occupation evolve during the training process.

## Discussion

The present study provides one of the first systematic, quantitative analyses of the career motivations and retention intentions of healthcare apprentices in a rural, peripheral region of Germany. The findings paint a more nuanced picture than public discourse on workforce shortages often suggests. They challenge common narratives while simultaneously identifying specific, yet often overlooked, levers for more sustainable workforce retention. In what follows, each result is read against the empirical evidence and the three theoretical lenses introduced in Section “Conceptual framework” — Self-Determination Theory, Social Cognitive Career Theory, and the push–pull framework — so that the patterns observed in this single rural setting can be situated within the broader literature rather than standing as isolated descriptive findings. The discussion below is structured along the four research questions introduced in Section “Introduction”.

### Career-choice motivations (RQ1)

The motivational hierarchy for career choice is most coherently read through Self-Determination Theory. The factors apprentices rank as decisive — personal interest, prior internship experience, and proximity to home, together with the instrumental consideration of income — are those that SDT treats as autonomous or instrumentally grounded in the person’s own situation, whereas the externally mediated channels of media exposure, career counselling, and parental advice, which SDT classifies as controlled influences, occupy the lowest tiers (Sections “Motivational influences on career choice”, Fig. 1; [9, 38]). This is consistent with the SDT-derived expectation that autonomous regulation, rather than externally administered guidance, sustains durable vocational commitment [16], and it converges with Koob and Tomic’s [26] finding that the perceived personal significance of healthcare work is positively associated with work engagement among German nursing trainees. It is likewise compatible with the broader retention literature that ties career entry and professional stability to personal competencies, task integration, and social appreciation [35, 40, 44] rather than to the externally projected image of the profession.

Within this single-institution cross-sectional sample, this pattern calls into question the leverage of the image-campaign approach that has long featured in German nursing recruitment [3]: the channels such campaigns operate through rank lowest, while authentic work exposure and the resolution of spatial and financial constraints rank highest. The distinctive intermediate position of parental advice, together with its loading on the pragmatic rather than the institutional factor in the exploratory factor analysis, refines this picture: apprentices appear to file parental influence with personal and family circumstances rather than with formal guidance, while the loading of prior internship on the institutional factor suggests that they construe placements as organised gateways arranged by schools or employers rather than as purely personal initiatives. This has direct implications for the allocation of public and institutional resources. Rather than investing in costly, large-scale image campaigns of questionable impact, funds are more plausibly channelled towards the enabling conditions — accessible, well-organised placements and the mitigation of spatial and financial barriers — that the autonomous-motivation account identifies as decisive.

### Internship and accessibility (RQ2)

Two contextual factors proved relevant beyond entry-stage motivation: prior internship experience and the geographic accessibility of the training institution. Both are most naturally interpreted through the contextual branch of Social Cognitive Career Theory, which formalises how proximal environmental supports and barriers shape career entry and persistence [29, 30]. Accessibility operates here as exactly such a proximal barrier: in a dispersed rural training market, commuting burden and the reachability of school and placement sites are not peripheral conveniences but conditions that can gate participation. The sub-group analyses locate this salience most strongly among younger apprentices and those with prior internship experience or family ties to healthcare, rather than as a uniform effect (Section “Internship experience and geographic accessibility”). This aligns with the rural-retention literature, in which local labour-market thinness and the perceived reachability of post-training career options are decisive [11, 25], and with review evidence that younger, early-career nurses are more mobile and less attached to their organisations than older colleagues [35]. Prior internship, in turn, functions in SCCT terms as a formative learning experience that supplies the direct exposure on which self-efficacy and realistic outcome expectations are built. The policy reading follows: investments in public transport, shuttle services, or mobility allowances, and in accessible structured placements, address the very contextual barriers SCCT identifies as constraining entry, and are likely to return more than recruitment messaging that leaves those barriers untouched.

### Training quality and retention (RQ3)

The regression analyses indicate that the strongest correlates of attrition intention are not primarily sociodemographic characteristics but the quality of the training processes themselves: poor ratings of school-based curricular content are associated with a higher intention to leave both the occupation and the sector, and dissatisfaction with company workflow organisation with a higher intention to leave the specific occupation (Table 5). Within Social Cognitive Career Theory this is the expected signature of the learning-experience pathway: the school- and company-based components of training are the proximal inputs through which vocational education either builds or erodes the self-efficacy beliefs and outcome expectations that ultimately feed persistence [29, 30]. The finding also gives an empirical location to a problem long discussed in the German vocational-nursing literature: the difficulties of the school–work interface, arising from divergent learning objectives and inconsistent competency recognition across learning venues [17, 33, 50]. Consistent with the domain profiles in Section “Practical experiences and orientation”, course organisation and curricular content emerge as the weakest-rated school-based elements, while company workflow and on-the-job instruction form the comparative weak points on the firm side; by contrast, instructors, classroom climate, and classmates on the school side, and practical content, trainers, and the social climate among trainees and co-workers on the company side, form coherently better-rated tiers. The company-workflow weakness is of particular practical relevance because it concerns the organisation of the firm-based training process, a lever training providers can directly act upon, and it echoes this literature’s emphasis on supportive onboarding and on accommodating heterogeneity in apprentices’ prior knowledge [10, 17]. The frequently invoked theory–practice gap thus appears here not as a rhetorical figure but as a measurable correlate of dropout intention, redirecting reform attention from post-training incentives such as retention bonuses towards the core processes of dual training itself.

### Sector vs. occupation retention (RQ4)

The most consequential finding qualifies the prevailing public narrative of pervasive dissatisfaction and impending attrition: a large majority of apprentices intend to remain in the healthcare sector over the long term (Sections “Determinants of future retention intentions”, Fig. 6), pointing to a substantial and possibly underestimated retention potential that debate focused on attrition statistics risks overlooking. The contrast with the sizeable share of qualified German nurses reporting no firm commitment to staying in the profession [44] is instructive: commitment appears comparatively intact at the training stage, marking early careers as the window in which retention policy has the most to preserve. At the same time, apprentices clearly distinguish the two retention horizons, expressing a somewhat higher willingness to remain in the sector than in their specific occupation. This gradient is most productively read through the push–pull framework [12, 28, 53]: occupation-specific dissatisfaction can act as a push away from a particular role, while sector-level pull, exerted through adjacent occupations, retains the person within healthcare. The reading is reinforced by Drange and Ingelsrud’s [11] evidence that limited career options and part-time conditions operate as push factors driving licensed practical nurses out of a specific care occupation while substantial numbers remain attached to the wider field. The regression results sharpen this picture: beyond curricular content, two company-side relational dimensions — on-the-job instruction and relations with co-workers — predict sector-level retention but not occupation-level retention (Table 5), suggesting that the day-to-day social and instructional fabric of the firm anchors apprentices to the wider sector even when commitment to a specific occupation wavers. This is coherent with retention research foregrounding social integration and the perceived availability of post-training career options as drivers of staying [10, 25]. For workforce planning the implication is that not every departure from a specific occupation represents a loss to the sector, and that policies promoting permeability and horizontal career pathways within healthcare can convert this latent flexibility into realised retention.

### Limitations

In interpreting these results, the study’s limitations must be considered. The sample is drawn from a single, specific rural region (Harz) and is therefore not representative of Germany as a whole. The generalisability of the findings must be tested in future studies with a multi-regional design. Furthermore, as a cross-sectional study, it can only suggest, not prove, causal relationships. The questionnaire also included ten open-ended items that are not analysed in the present manuscript; their systematic evaluation requires qualitative coding procedures beyond the scope of the present paper and will be reported in a dedicated follow-up study. This qualitative material is expected to contribute in particular to understanding the direction of change in occupational perceptions — which the closed-format item did not capture — and to contextualising the mechanisms behind specific quantitative findings.

## Conclusion

This study offers systematic, quantitative insights into the career motivations, early training experiences, and retention intentions of apprentices in nursing and related healthcare professions in a rural German context — an area that has received limited empirical attention despite its policy relevance. Our findings challenge the prevailing narrative of generalized disillusionment: the vast majority of respondents envisage a future in healthcare, yet they differentiate clearly between remaining in a specific occupation and remaining within the sector. This nuance underscores the potential of targeted interventions that focus not only on entry into training, but on maintaining flexible career pathways within the healthcare system.

The analyses identify concrete levers for improving the sustainability of vocational training. Curricular content in vocational school emerges as a significant determinant of both retention intentions and changes in occupational perceptions, while workflow organisation in the firm-based component is a comparable determinant of occupational retention. Ex-ante transport considerations and the day-to-day company environment further shape early access and motivation. These findings point to actionable opportunities for training providers, regional planners, and policymakers: raising the quality and coherence of school content, supporting companies in optimising their internal training and workflow processes, and coupling rural training strategies with mobility support. Embedding project-based learning phases with rotating practical emphases — akin to clinical rotations but with integrated theory–practice consistency checks — could further bridge the gap between classroom and workplace learning that our regression analyses identified as a key risk factor.

While the data stem from one rural region, they highlight mechanisms likely to be relevant across comparable contexts facing similar demographic and geographic challenges. Expanding such evidence-based approaches beyond single districts, and following apprentices longitudinally into their early careers, represents a crucial next step for strengthening the healthcare workforce in the face of demographic change. Only through such systematic investigation can we move beyond well-intentioned but empirically ungrounded interventions toward evidence-based strategies that sustain both individual career pathways and sectoral workforce needs.

## Supplementary Information

Below is the link to the electronic supplementary material.

Supplementary Material 1

Supplementary Material 2

Supplementary Material 3

Supplementary Material 4

Supplementary Material 5

Supplementary Material 6

## Data Availability

The datasets generated and analyzed during this study are not publicly available due to privacy protection of study participants but are available from the corresponding author upon reasonable request. Aggregated data sufficient to replicate the reported findings without enabling participant reidentification can be provided.
