# Supplementary material for: Career choice, training satisfaction, and retention intentions among healthcare apprentices: a cross-sectional study in rural Germany
Source: BMC Health Serv Res. 2026 Jul 7;26:937. doi: 10.1186/s12913-026-15022-y (PMC13352847; doi:10.1186/s12913-026-15022-y)
Supplement: Supplementary file 1 — Supplementary Material 1 [file 12913_2026_15022_MOESM1_ESM.docx]

# Questionnaire

The survey comprised nine sections with a total of 25 questions, administered in German and translated here into English. Response options are indicated in square brackets. Variable names used in the regression analyses (Tables 4 and 5) are indicated in parentheses where applicable.

**Section 1: Motivation for choosing apprenticeship**

**1.1** How important were the following factors in choosing your current apprenticeship?
Response scale: 1 = very important, 2 = rather important, 3 = unsure/unclear, 4 = rather unimportant, 5 = completely unimportant

- Media exposure (e.g., social media, TV films/documentaries/series, newspapers)
- Career counselling (e.g., counselling sessions at school)
- Internship experience (e.g., experiences from school internships) (Prior internship)
- Parents/relatives (e.g., conversations or recommendations from family)
- Income prospects (salary prospects in the profession)
- Proximity to home (proximity of training place to residence)
- Personal interest (personal interest in the professional field)

**1.2** What motivated you to choose your current apprenticeship? [Open text response]

**Section 2: Changes in perception of the profession**

**2.1** Has your perception of the profession changed since you decided on your current apprenticeship? (Change in perceptions)
Response scale: 1 = changed very strongly, 2 = changed rather strongly, 3 = unsure/unclear, 4 = changed rather little, 5 = not changed at all

**2.2** Please tell us what has changed in your perception of the profession. [Open text response]

**2.3** Was there something that surprised you about the profession – something that was completely different from what you had thought before starting the training? [Open text response]

**Section 3: Career orientation and internships**

**3.1** Did you complete an internship for career orientation? (Prior internship) [Yes/No]

**3.2** If you remember your internship(s), what was good and what was less good from today's perspective? [Open text response]

**3.3** As a young person, one usually doesn't know many companies where internships are possible. How did you find the position(s) for your internship(s)? [Open text response]

**3.4** Did transport connections play a role in your decision for the apprenticeship or internship?
Response scale: 1 = yes, transport connections were very important, 2 = rather yes, transport connections were important, 3 = unsure/unclear, 4 = rather no, transport connections were less important, 5 = no, transport connections were completely unimportant

**3.5** Please tell us what was difficult or special regarding mobility or transport connections in your career choice or career orientation. [Open text response]

**Section 4: Ideas for improving career orientation**

**4.1** What do you think students should absolutely know to orient themselves well about professions? [Open text response]

**4.2** Do you have suggestions or ideas on how to better motivate young people for nursing professions while still in school? [Open text response]

**Section 5: Training evaluation**

**5.1** Looking at your current training: What works well, and what could be improved?
Response scale: 1 = works very well, 2 = rather well, 3 = unsure/unclear, 4 = rather poorly, 5 = works very poorly

**School-based training:**

- Workflow/organisation of training (School: Workflow)
- Type of guidance and explanations (School: Didactic guidance)
- Training content (School: Content)
- Instructors (School: Instructors)
- Treatment of apprentices (School: Climate)
- Colleagues and type of collaboration (School: Classmates)

**Company-based training:**

- Workflow/organisation of training (Company: Workflow)
- Type of guidance and explanations (Company: On-the-job guidance)
- Training content (Company: Practical content)
- Mentors/trainers (Company: Trainers)
- Treatment of apprentices (Company: Climate among apprentices)
- Colleagues and type of collaboration (Company: Coworkers)

**5.2** Regarding your training – what works well and what could be better? [Open text response]

**Section 6: Future career intentions**

**6.1** Do you think the profession you are currently training for will still be suitable for you in the future? How likely do you think you will still be working in this field in the next 5 years?
Response scale: 1 = very likely, 2 = rather likely, 3 = unsure/unclear, 4 = rather unlikely, 5 = very unlikely

- I will remain in the occupation I'm training for (Retention: occupation)
- I will remain in the professional sector (e.g., healthcare sector, hospital or nursing home) (Retention: sector)

**6.2** Generally speaking, what would be possible reasons for you to become uncertain about continuing in your profession? [Open text response]

**6.3** Do you intend to relocate to another region in the next 5 years (change of residence more than 30 km)? (Planned relocation)
[Yes, I intend to move to another region / No, I currently do not plan to move to another region / Other]

**Section 7: Demographics**

**7.1** Age (Age) [<18, 18-20, 21-25, 26-30, 31-35, >35]

**7.2** Gender (Sex) [Male/Female/Diverse]

**7.3** Highest educational qualification (School degree)
[Lower secondary / Intermediate secondary / Upper secondary (Abitur) / Other]

**7.4** Did you have work experience before your current training (e.g., mini-job, part-time or full-time)? (Prior work experience) [Yes/No]

**7.5** Are there people in your immediate family working in nursing or social care? (Relatives in healthcare) [Yes/No]

**Section 8: Living environment and transport connections**

**8.1** How would you describe your residential environment? (Residence)
[Urban or large urban area / Small town or larger village, but still near a city / Very rural area]

**8.2** What is your commute distance for training? (Please indicate for both locations)

- Residence – Training company: Duration in hours/minutes (sum of round trip) (Daily commute time: workplace)
- Residence – Vocational school: Duration in hours/minutes (sum of round trip) (Daily commute time: school)
- Means of transport used [Open text response]
- Were/are there difficulties with the route? (e.g., connection problems, delays) [Open text response]

**8.3** How stressful do you find the commute in connection with your training?
Response scale: 1 = very stressful, 2 = rather stressful, 3 = unsure/unclear, 4 = rather not stressful, 5 = not stressful at all

- Residence – Training company
- Residence – Vocational school

**Section 9: Final comments**

**9.1** Finally, we would like to give you the opportunity to provide us with general comments, suggestions or remarks. Any comments (about training, career orientation or the survey) are welcome. [Open text response]
